# Simulation Modeling Unveils the Unalike Effects of Alternative Strategies for Waterbird Conservation in the Coastal Wetlands of Sardinia (Italy)

**DOI:** 10.3390/biology12111440

**Published:** 2023-11-16

**Authors:** Alessandro Ferrarini, Marco Gustin, Claudio Celada

**Affiliations:** Lipu-BirdLife Italy, Via Pasubio 3, 43122 Parma, Italy; marco.gustin@lipu.it (M.G.); claudio.celada@lipu.it (C.C.)

**Keywords:** alpha diversity, central eastern Mediterranean flyway, counterfactual scenarios, management scenarios, Natura 2000 sites, non-parametric Bayesian network, species conservation, waterbirds, wetland traits

## Abstract

**Simple Summary:**

Natural wetlands supply food and shelter for many bird species and offer stopover sites that allow waterbirds to make long migratory journeys. Human impacts and ongoing climate change are however reducing the ability of wetlands to provide such essential services to avian diversity. Therefore, scientists are now forced to consider new approaches to the proactive management of remaining marginal wetlands so as to preserve the associated biodiversity as well. Because regional planning and wetland management are often debatable issues, we argue here that in silico simulation modeling, combined with field surveys and data collection, provides an appropriate methodology to address this matter effectively. Modeling and dynamically simulating the changes expected to the levels of avian diversity as a function of plausible counterfactual and management scenarios provides an informed and scientifically defensible basis for proactive conservation strategies. Such simulations present an opportunity to test numerous different scenarios, exploring their implications for the conservation of avian diversity, thus providing a well-grounded and realistic approach to dealing with inherent uncertainties in wetland management and waterbird conservation.

**Abstract:**

The Sardinian wetlands (Italy) act as stopover sites for many migratory birds along the central eastern Mediterranean bird flyway. These wetlands are now severely threatened by human activities and climate change. Accordingly, we built a simulation framework to predict the effects of several counterfactual and management scenarios on the level of avian diversity in the coastal wetlands of Sardinia. We found that the alpha avian diversity (i.e., the mean number of avian species per wetland) is destined to (a) decrease due to the most likely increase in water salinity, water discharges, and tourism pressure; and (b) halve (from 14.9 to 7.4, with 9 wetlands out of 22 predicted to host only between two and five waterbird species) in the worst possible scenario. However, the results also showed that proper management strategies could prevent and reverse such outcomes. Restrictions on tourism activities, water desalination, prevention of future saltwater intrusions, and the prohibition of water discharges could markedly favor the avian diversity in these wetlands, with an expected increase in the alpha avian diversity from 14.9 to 24.8 (and 10 wetlands out of 22 predicted to host from 29 to 32 waterbird species) in the best possible scenario. The importance of our results could be emphasized in the management plans of these important wetlands, most of which belong to the Natura 2000 network.

## 1. Introduction

The European Union (EU) is particularly concerned with the protection of biodiversity [1,2]. This has led to the establishment of more than 26,000 Natura 2000 sites covering about 26% of the land and 11% of the seas in the EU [3] under the two main directives governing the creation and management of these sites: the Birds (2009/147/EC) and the Habitats (92/43/EEC) Directives. According to the European Green Deal [4], the EU’s Biodiversity Strategy for 2030 proposes to expand the Natura 2000 and protected areas network to at least 30% of Europe’s land and sea areas [5]. The basis for the conservation of species and habitats within Natura 2000 sites is the management plan that provides the legal foundation for applying the conservation measures [6].

**Figure 1:**
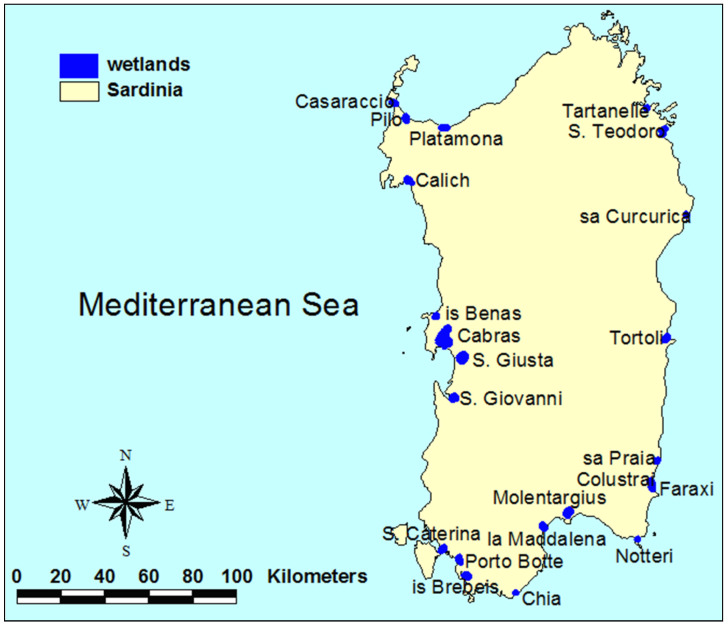
Study area (Sardinia, Italy). The total surface area of the 22 wetlands under study is 5545 ha, and the average inter-distance is 12.6 km. With the exception of Tartanelle and Tortoli, all wetlands belong to the Natura 2000 network.

The Sardinian wetlands lie along the Sardinia–Corsica corridor of the central eastern Mediterranean bird flyway. These wetlands act as stopover sites for migratory waterbirds and support waterbirds that do not migrate and thus are important during the entire annual cycle [7]. In summer (July–September), the Sardinian wetlands host 59 bird species, of which almost 90% are migratory species whose conservation interest is primarily at European and global level [8]. The purple heron *Ardea purpurea*, the slender-billed gull *Larus genei*, the black-winged stilt *Himantopus himantopus*, the greater flamingo *Phoenicopterus roseus*, and the little tern *Sternula albifrons* are examples of species of high conservation interest (Annex I of the Birds Directive) in these wetlands [9]. However, tourism pressure, water salinization, water discharges, and elevated water depth are now affecting many waterbird species in the Sardinian wetlands, especially in summer [9,10]. Accordingly, in this study, we built a simulation framework to predict the effects of several counterfactual and management scenarios on the level of avian diversity in the coastal wetlands of Sardinia (Figure 1; 22 wetlands, of which 20 belong to the Natura 2000 network). Our results could be integrated into the management plans of these important wetlands by making good use of the opportunities provided by the recent EU legislation on this issue.

## 2. Materials and Methods

### 2.1. Field Surveys

During July–September 2016, for each wetland, we performed five sampling sessions of avian diversity and wetland traits at regular intervals of 10–15 days between sessions. We used regularly spaced sampling points with a 200 m minimum distance in order to minimize spatial autocorrelation [11]. We collected 144 sampling points, where we employed the standard point count sampling method [12] that required a 100 m observation distance around each sampling point and a 15 min observation time with a recording of all visual contacts. In addition to the avian diversity, we also assigned nine traits to each wetland (Table 1). We used the GoogleEarth™ images to evaluate wetland size. Isolation was computed as the boundary-to-boundary distance from the nearest wetland, and distance to the coastline as the minimum distance of wetland boundary from the coastline. We measured the water level at each sampling point by using a metric rod and then averaged over the five dates of sampling. Salinity was evaluated indirectly through the frequency of sea-water intrusions during field surveys. Water diversions and discharges quantified the presence of active drainage and discharge systems, respectively. Tourism pressure and anthropization measured the intensity of tourist activities (walking, horse riding, water sports, angling, etc.) and the presence of anthropic elements (greenhouses, dumpsites, quarries, camping sites, caravan parks, etc.) in the surroundings of each wetland, respectively. These latter variables were evaluated in a semi-quantitative way (Table 1) because an alternative approach based on precise assessment was outside the reach of this study. While quantitative values are preferable, semi-quantitative scores could adequately discriminate between the different levels of these variables in the studied wetlands. Field surveys were carried out by the same group of experts, thus assuring a well-founded comparative assessment of these variables among wetlands.

### 2.2. Model Setup and Validation

**Figure 2:**
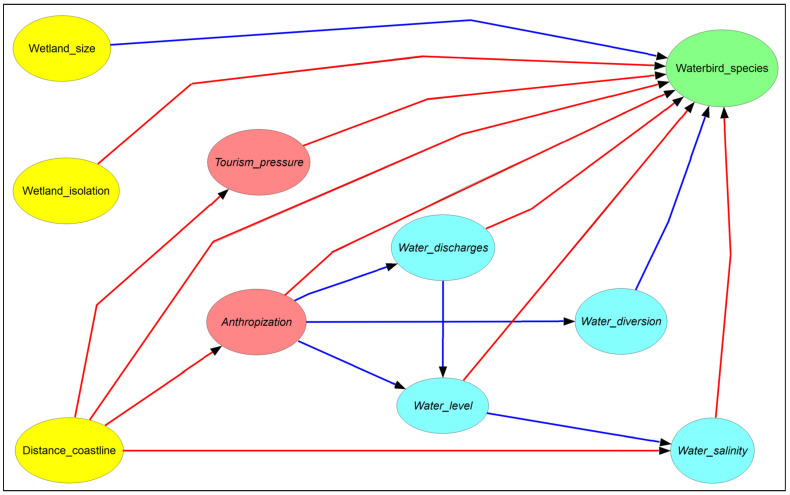
The conceptual model (expert knowledge) of the level of avian diversity (green ellipse) in the Sardinian wetlands as a function of spatial (yellow ellipses), anthropic (red ellipses), and hydrological (blue ellipses) variables. Arrows denote the hypothesized direct influences of the source variables upon the destination variables. Blue and red arrows indicate positive and negative effects, respectively.

First, we built a conceptual model (Figure 2) of the alpha avian diversity (i.e., the mean number of avian species per wetland) in the study area on the basis of our previous studies [8,9,10]. The aim of this step was to provide a visual framework of how the drivers (e.g., salinity and mean water level) influence each other and the target variable (alpha avian diversity). The conceptual model was useful for mimicking the causal chains that determine the levels of alpha avian diversity in the studied wetlands.

Secondly, we translated the conceptual model into a non-parametric Bayesian hierarchical network (NBHN; [13,14,15,16]). An NBHN is a directed acyclic graph where a set of variables (nodes) represent states (e.g., tourism pressure) of a system (the 22 wetlands under study), and a set of directed links (arcs) represent conditional (partial) correlations between the nodes. The NBHN used in this study was thus an interacting network comprising all the distal and proximal variables that are expected to rule the alpha avian diversity in the study area. 

The NBHN can deal with both discrete (as long as variables are defined in an ordinal scale) and continuous marginal distributions. Changes to each variable produce direct and indirect effects on all other variables, whose strength depends on conditional (partial) correlations. With the model structure in place (Figure 2), the marginal distributions of the variables and the conditional correlations between nodes were calculated (i.e., model calibration) using the empirical data available from our field surveys. From a mathematical viewpoint, the nodes represent univariate random variables (*X*_1_, *X*_2_,…, *X_n_*), and the conditional correlations are calculated using the normal copula [17]. By using Sklar’s theorem [18], any joint cumulative distribution function (here denoted *F*_1_…*F_n_*) of variables *X*_1_…*X_n_* can be rewritten as a function of the corresponding copula *C*:

*F*_1...*n*_ (*X*_1_...*X_n_*) = *C*(*F*_1_(*X*_1_)...*F_n_*(*X_n_*))

where *F_i_*(*X_i_*) is the marginal distribution of the *i*-th variable. The normal copula is expressed as:

*C_ρ_*(*u_1_*...*u_n_*) = *Φ_R_*(*Φ^−1^*(*u_1_*)...*Φ^−1^*(*u_n_*))

where *Φ* is the standard normal distribution, *Φ^−^*^1^ denotes its inverse, and *Φ_ρ_* is the bivariate Gaussian cumulative distribution with conditional correlation *ρ* between the two marginal uniform variables *u* and *v*. 

Thirdly, the model validation required testing whether normal copulas adequately represented the original data. Two determinants (*D*) had to be computed [13,14,15,16]: the *D_ER_* (determinant of the empirical rank correlation matrix; i.e., the dependence structure of the original data) and the *D_NR_* (determinant of the empirical normal rank correlation matrix), calculated as

$$D=\prod_{i,j}(1-\rho^{2}{}_{ij})$$

where *ρ* is the partial correlation assigned to the arc connecting nodes *i* and *j*, and the product is taken over all arcs in the NBHN. *D* varies in the [0, 1] interval, reaching 1 if all variables are independent, and 0 in case of multivariate linear dependence. This validation step was carried out by simulating (i.e., 10^4^ simulations) the sampling distribution of *D_NR_* and checking whether *D_ER_* was within the 90% confidence band of *D_NR_*; if so, the normal copula assumption could not be rejected at the 10% significance level [16]. The empirical rank correlation matrix was calculated by using Spearman’s *rho* correlation coefficient

$$rho=1-(6*\sum_{i=1}^{n}d_{i}{}^{2}/(n^{3}-n))$$

where *n* is the number of wetlands, and *d_i_* is the rank of the *i*-th wetland in the first variable minus the rank of the *i*-th wetland in the second variable.

In order to apply the NBHN to our field data, we used the UninetEngine package [13]. Recent applications of the NBHN modeling to ecological and environmental systems are found in [19,20,21,22,23].

### 2.3. Baseline, Counterfactual, Management, and Mixed Scenarios

After the model was validated positively, the counterfactual and management simulations were carried out by conditionalization, i.e., by setting the value of one or more variables of the NBHN to evaluate how it/they change the state of other variables and, in particular, the target variable (i.e., alpha avian diversity). 

The baseline scenario (Table 2) simply represents the distribution of the alpha avian diversity in the 22 wetlands in 2016 and was the reference point against which the other scenarios could be compared. The counterfactual scenarios (Table 2) simulate the effects on avian diversity of the expected trends of the variables considered in the NBHN (i.e., if no conservation measures happen). In other words, the counterfactuals represented worst-case scenarios because previous studies showed that human/climate threats are most likely to increase in the near future in the study area [8,9,10]. We thus simulated the generalized increase in tourism pressure, water salinity, water discharges, mean water level, and decrease in water diversions. In the studied wetlands, the mean water level (often resulting from artificial regulation for human activities, like angling and fish farming) is too high for many waterbird species, which disadvantages primarily small waders, species feeding on invertebrates, and trans-Saharan migrants [9]. That is why the increase in mean water level and decrease in water diversions were considered worst-case scenarios. We also simulated the worst possible scenario, where all these impacts act together upon the avian diversity of the Sardinian wetlands. On the contrary, the management scenarios (Table 2) typify here the best-case assumptions, where some conservation measures counteract the expected trends of the variables influencing the avian diversity. Because the NBHN simulations revealed that the highest values of alpha avian diversity occurred for “mean water level = 3” (i.e., between 20 and 30 cm), the NBHN was conditioned by this value in the management scenarios. We also simulated the best possible scenario, where conservation measures counteract all variables impacting avian diversity. The mixed scenarios (Table 2) are halfway between the counterfactual and management scenarios, where in fact all the conditions deteriorate except for one that is neutralized by conservation measures.

## 3. Results

**Figure 3:**
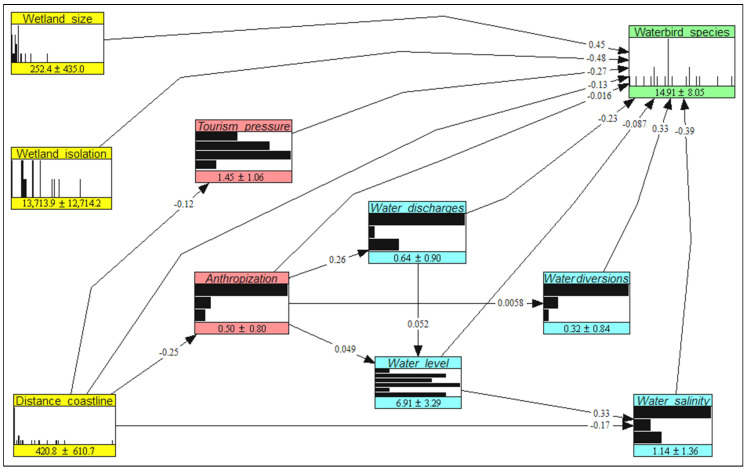
The estimated non-parametric Bayesian network used to predict the effects of counterfactual, management, and mixed scenarios on the level of avian diversity in the Sardinian wetlands.

The NBHN result is shown in Figure 3. The node colors have the same meaning as those in Figure 2. The nodes are presented as histograms, with numbers indicating the means and standard deviations of the variables. Values on the arcs are partial correlation coefficients between variables.

**Figure 4:**
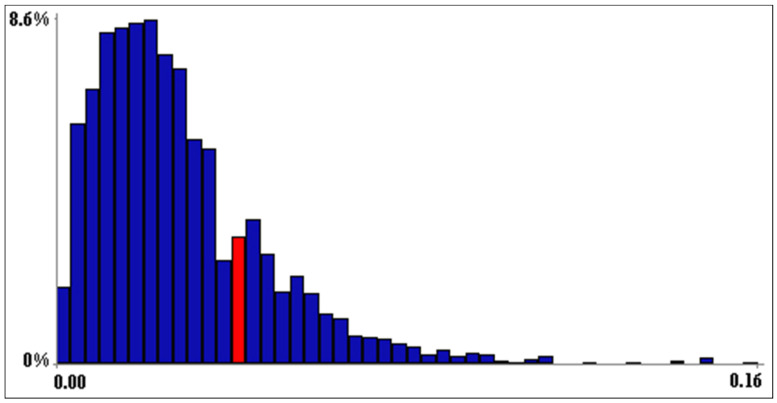
Model validation. The determinant of the empirical rank correlation matrix (*D_ER_*) fell between the 0.75 and 0.80 quantiles (red bin) of the confidence band of the empirical normal rank correlation matrix (*D_NR_*).

The NBHN model was successfully validated (i.e., the partial correlation matrix under the normal copula assumption was a satisfactory approximation of the partial correlation matrix of the original data); in fact, *D_ER_* fell within the 90% confidence band of *D_NR_* (Figure 4).

**Figure 5:**
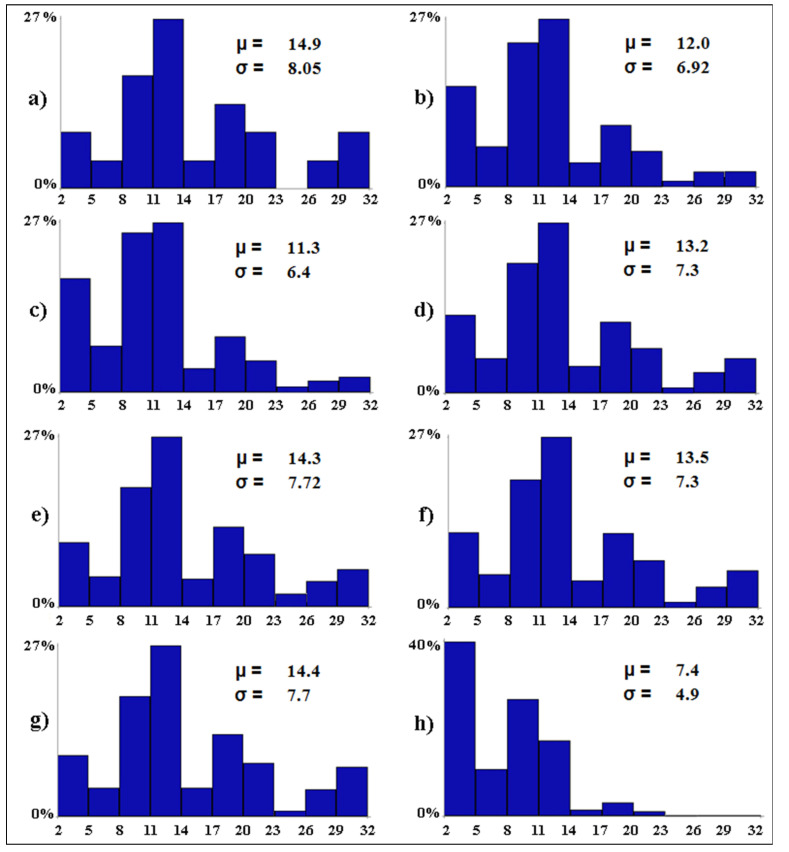
Baseline (**a**) and worst-case (**b**–**h**) scenarios. The letters associated with the scenarios have the same meaning as per Table 2. On the *x*-axis, the expected distribution of the avian diversity (expressed as number of bird species in 10 equal-size intervals) is reported. On the *y*-axis, the proportion (in %) of wetlands that are expected to fall into each range of avian diversity is shown. For each scenario, the Greek letters indicate the mean (*µ*, i.e., alpha diversity) and standard deviation (*σ*) of the expected number of bird species per wetland.

The worst-case scenarios (Figure 5) show the (expected) elevated impact of water salinity on the alpha avian diversity (i.e., the mean number of bird species per wetland). All other variables being equal, if water salinity becomes widespread in all wetlands (scenario *c*), the alpha avian diversity is expected to decrease by 3.6 species. Tourism pressure was the second most important type of impact on the waterbirds. Ceteris paribus, in case tourism pressure becomes widespread in all wetlands (scenario *b*), the alpha avian diversity is expected to decrease by 2.9 species. The alpha avian diversity of these wetlands is expected to halve (from 14.9 to 7.4) in the worst possible scenario, with 40.9% of the wetlands (i.e., 9 out of 22) predicted to host only between two and five waterbird species (scenario *h*).

**Figure 6:**
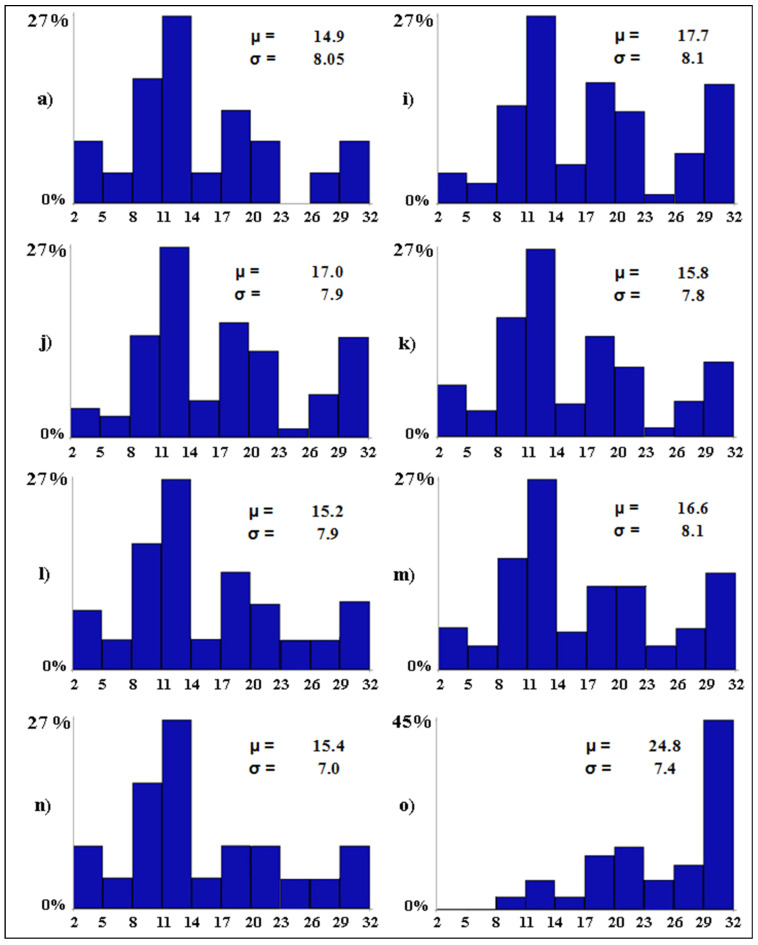
Baseline (**a**) and best-case (**i**–**o**) scenarios. The letters associated with the scenarios have the same meaning as per Table 2. On the *x*-axis, the expected distribution of the avian diversity (expressed as number of bird species in 10 equal-size intervals) is reported. On the *y*-axis, the proportion (in %) of wetlands that are expected to fall into each range of avian diversity is shown. For each scenario, the Greek letters indicate the mean (*µ*, i.e., alpha diversity) and standard deviation (*σ*) of the expected number of bird species per wetland.

The best-case scenarios (Figure 6) depict the importance of three conservation measures. All other variables being equal, if tourism pressure becomes null in all wetlands (scenario *i*), the mean number of bird species per wetland is expected to increase by 2.8 species. Water desalination was the second most important conservation measure. Ceteris paribus, in case water salinity becomes null in all wetlands (scenario *j*), the alpha avian diversity is expected to increase by 2.1 species. Setting the water levels between 20 and 30 cm in all wetlands would lead to an average increase of 1.7 species per wetland (scenario *m*). The alpha avian diversity of the studied wetlands is expected to increase by 66.4% (from 14.9 to 24.8) in the best possible scenario, with 45.4% of the wetlands (i.e., 10 out of 22) predicted to host from 29 to 32 waterbird species (scenario *o*).

**Figure 7:**
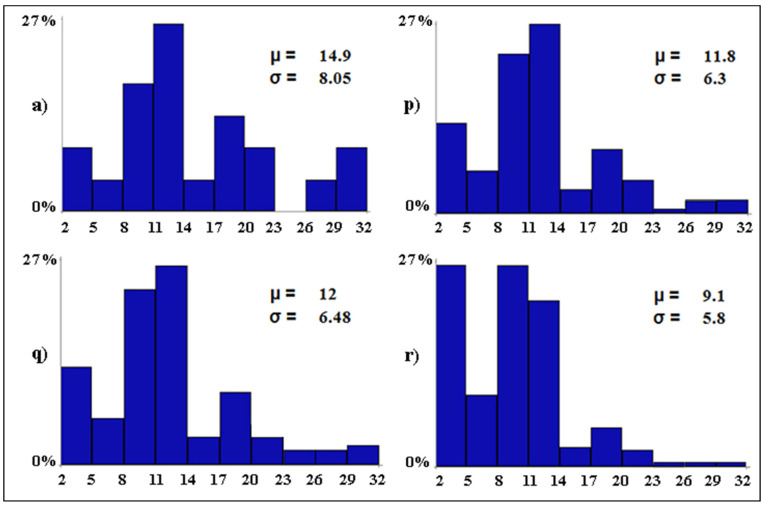
Baseline (**a**) and mixed (**p**–**r**) scenarios. The letters associated with the scenarios have the same meaning as per Table 2. On the *x*-axis, the expected distribution of the avian diversity (expressed as number of bird species in 10 equal-size intervals) is reported. On the *y*-axis, the proportion (in %) of wetlands that are expected to fall into each range of avian diversity is shown. For each scenario, the Greek letters indicate the mean (*µ*, i.e., alpha diversity) and standard deviation (*σ*) of the expected number of bird species per wetland.

The mixed scenarios (Figure 7) show that one single conservation measure is not able to preserve the baseline levels of avian diversity in case all other variables deteriorate. In fact, if restrictions on tourism activities are the only conservation measure while all other variables decline, the mean number of bird species per wetland is expected to decrease by 3.1 species (scenario *p*). Water desalination alone could only limit the mean loss of species per wetland to 2.9 (from 14.9 to 12, scenario *q*). The prohibition of water discharges alone would be largely insufficient as the mean loss of species per wetland would be 5.8 (from 14.9 to 9.1) with 27.3% of the wetlands (i.e., 6 out of 22) predicted to host only between two and five waterbird species (scenario *r*).

## 4. Discussion

Numerous studies have documented the adverse effects of human/climate threats on wetlands and the associated waterbird species worldwide [24,25,26,27,28,29,30,31,32,33,34,35]. Accordingly, in this study, we developed a simulation modeling framework, combined with field surveys and data collection, to provide an informed and scientifically defensible basis for proactive wetland management and waterbird conservation. Our decision tool detected how the influencing variables regulate the levels of alpha avian diversity in the studied wetlands, and how changes to these variables can alter such diversity. 

We found that if no conservation measures are realized in the coastal wetlands of Sardinia, then the situation for the waterbird species will become critical. Following the recent trends of the anthropic and hydrological variables in these wetlands [8,9,10], the mean number of species per wetland would decrease due to the most likely increase in water salinity, water discharges, and tourism pressure, and would halve (from 14.9 to 7.4, with 9 wetlands out of 22 predicted to host only between two and five waterbird species) in the worst possible scenario (i.e., if all the influencing variables deteriorate). However, our results can also ignite optimism about the conservation of the waterbird species. In fact, proper management strategies could markedly prevent and reverse such negative outcomes, up to the best possible scenario where the alpha avian diversity is expected to increase from 14.9 to 24.8, and 10 wetlands out of 22 are predicted to host from 29 to 32 waterbird species. 

### 4.1. Model Properties and Assumptions

The NBHN approach used here made it possible to (a) formulate a clear working hypothesis on the network of relevant and interacting variables that actually determine the alpha avian diversity in the coastal wetlands of Sardinia, (b) build a custom network-like model to represent and test this hypothesis, (c) validate or reject the hypothesis by comparing empirical (i.e., data-based) correlation structures with correlation structures of the non-parametric Bayesian hierarchical network. This approach showed several advantages when compared against other methods, for example: (a) its graphical nature made the dependence configuration explicit, (b) it can deal with ordinal (semi-quantitative) variables, and allow for hierarchical network structure, which cannot be achieved through regression methods, and (c) it allowed us to split the overall influences among variables into direct and indirect influences, which in turn provided the opportunity to dynamically simulate changes to any variable and predict the direct and indirect effects expected for the target variables (avian diversity).

The anthropic, hydrological, and avifaunal data used in this study were sampled in summer (July–September). Therefore, the results of our study are pertinent to the summer period only. The rationale is that summer behaves as the bottleneck period of the year for wetlands and the associated biodiversity due to the utmost increase in tourism activities, water discharges from tourism and recreational facilities, and anthropization level (illegal dumpsites, camping sites, caravan parks, and so forth) in the close surroundings of the studied wetlands [8,9,10]. In addition, as wetlands in Sardinia belong to the Mediterranean bird flyway, in summer, they host the highest number of avian species and individuals. Accordingly, we focused our study on the time interval that is most critical and important for waterbird conservation.

In this study, we used alpha diversity as a measure of bird species richness. Alpha diversity is the diversity in species at individual sites (e.g., wetlands, plots, quadrats, etc.), and is quantified by the mean number of species (i.e., mean species richness) present at the studied sites [36]. Two further measures of diversity are common in the scientific literature: gamma and beta diversity [37]. The former is the number of species present in the whole region of interest for the study (here, the overall number of avian species present in the 22 wetlands under study), while the latter is the variation in species composition among sites in the geographic area of interest. However, these different measures of diversity are not independent; in fact, beta diversity can be viewed as a measure that compares diversity at two different scales (alpha and gamma diversity). This comparison can be realized by using the classical multiplicative formulation (beta = gamma/alpha), additive partition (beta = gamma − alpha), or more complex approaches [38]. Accordingly, our methodological framework could be further extended to predict how changes to the level of alpha diversity can, in turn, alter gamma and beta diversity as well.

In our study, we used a bottom-up approach to define the priority threats to avian diversity. In our approach, the priority threats emerged from simulations of those changes to wetland traits that can lower the most the level of alpha avian diversity in the Sardinian wetlands. We are aware that top-down approaches exist as well (e.g., threat analysis and threat reduction assessments [39,40]), where panels of experts assess the regime attributes (extent, severity, and magnitude) of each human-induced disturbance to carry out an arrangement and quantification of the main threats and select the priority ones. However, we think that bottom-up simulation modeling can provide several advantages with respect to top-down approaches; for example, it shows that threats can be hierarchically nested and not independent (Figure 3), and also provides predictions of the direct and indirect effects of such threats upon biodiversity.

Our modeling and simulation framework involved also spatial variables (i.e., wetland size, isolation, and distance to the coastline) that were not used in the counterfactual, management, and mixed scenarios because these variables are not expected to change in the near future. This raises the question of why they were inserted into the NBHN model. The rationale is that these spatial variables largely influence the alpha avian diversity in the studied wetlands (see Figure 3), and thus their presence in the model allowed us to precisely determine the influence (i.e., partial correlation) of the anthropic and hydrological variables on the avian diversity, with the effect of the spatial variables removed. Without these spatial variables in the model, such partial correlations would have been spurious, and the simulations possibly biased.

The avian diversity of the Mediterranean wetlands could be subject to two further threats that were not mentioned in this study: bird predation by vagrant dogs [41,42] and nest destruction by human trampling [43]. The former was not detected during the five sessions of field surveys in the Sardinian wetlands; thus, we considered it as absent or sporadic in the studied wetlands. The latter is probably present in these wetlands, and it was indirectly accounted for by the variable “tourism pressure”. 

In this study, we investigated the effects of counterfactual and management scenarios on species presences–absences, rather than abundances. In fact, presence–absence data are less prone to errors during field surveys. We are aware that the effects of the best-case, worst-case, and mixed scenarios on avian diversity could be slow and affect initially only species abundances. However, we expect that, as wetlands traits increase (decrease) above (below) certain levels, the decline in species abundances will turn into disappearance, while the episodic presence of some species will become stable. To some degree, we also expect that favoring the presence of avian species by acting upon the wetland traits will benefit species abundances as well. For example, since 2014, the hydraulic interventions (construction and maintenance of levees, and water renewal to avoid stagnation) applied in the Molentargius wetland (Figure 1) have largely increased the local breeding population of the greater flamingo *Phoenicopterus roseus* (Sergio Nissardi, personal communication).

### 4.2. Implications for Waterbird Conservation

In the study area, tourism pressure was very elevated (widespread) at Calich, S. Teodoro, Tartanelle, and Tortoli, and elevated (scattered) at Chia, la Maddalena, Notteri, Pilo, Porto Botte, S. Giusta, and Sa Curcurica. Tourist activities and recreational activities (e.g., hunting and fishing activities, free camping, etc.) exert a considerable influence on wetland integrity and the associated avian diversity, including illegal hunting, intentional/unintentional removal of waterbirds, damage to nests, and noise disturbance, thus inducing reduced breeding success and altered habitat use [44]. 

Artificial water regulation, where several channels connected to the sea are used to maintain high water levels for human activities (angling and fish farming), is responsible for both the elevated levels of water depth and water salinity in these wetlands [8,9,10]. 

Water salinity was very elevated (widespread) at Calich, Colostrai, Feraxi, is Benas, and Sa praia, and elevated (scattered) at S. Teodoro, Sa Curcurica, and Tartanelle. Water salinity induces dehydration in birds, reduces the waterproofing of feathers, and alters thermoregulation, thus interfering with diving and flying [45]. Salinity also impacts negatively the vegetation of the wetland shoreline, which can serve as a resting and foraging habitat for several waterbird species [44]. Water desalination would require both the construction of artificial dune cordons to minimize saltwater intrusions and the closure of all channels that directly, and often unrestrainedly, connect these wetlands to the sea. Only in a few cases have these interventions been proposed in the management plans of these wetlands (e.g., Cabras, Porto Botte, and San Teodoro), but they have not been implemented yet.

Unlike the Sicilian wetlands where water shortage or complete drainage is very common in summer [46], in the Sardinian wetlands the mean water level was very elevated (>100 cm) in seven wetlands (Calich, Casaraccio, Feraxi, S. Giovanni, Sa praia, Tartanelle and Tortoli) and elevated (>50 cm) in further six wetlands (Cabras, la Maddalena, Platamona, Porto Botte, S. Caterina, S. Giusta). Elevated water levels highly disadvantage species feeding on invertebrates and, in particular, wading and dabbling birds that prefer shallow water to forage [47]. In addition, the artificial water regulation in these wetlands minimizes the water-level fluctuations that benefit avian diversity by providing more foraging opportunities [47]. 

Organic and chemical pollution due to water discharges from urban and farm areas and tourist facilities was elevated (scattered) at Cabras, Colostrai, Feraxi, is Benas, S. Giusta, and Tortoli. The decrease in water quality directly and indirectly affects the use of these wetlands by waterbirds. The consequences of organic and chemical pollution include modified habitat use and a decrease in reproductive success [48].

The remarkable differences in the levels of avian diversity between the worst (Figure 5h) and best (Figure 6o) possible scenarios call for effort and responsibility on the part of local administrations and stakeholders to better preserve and restore the coastal wetlands, and the associated avifauna, of Sardinia. Our results showed that one conservation measure alone cannot compensate for the worsening of all other influencing variables. Two joint management options seem highly feasible in these Natura 2000 sites: restriction of tourism activities in the close surroundings of the wetlands, and the prohibition of water discharges from the surrounding urban and farm areas and tourist facilities. Our modeling framework showed that the conjoint application of restrictions on tourism activities and the prohibition of water discharges would be enough to preserve the baseline levels of avian diversity in the studied wetlands. In fact, in the case where all the influencing variables become worse with the exception of tourism pressure and water discharges that become null, the NBHN simulations predicted the mean number of avian species per wetland to be 14.4 (±6.98 S.D.), which is almost equal to the baseline diversity value 14.9 (±8.05 S.D.). However, this conservative management scenario is not particularly desirable because the environmental conditions of these wetlands in the baseline scenario have already deteriorated due to human impacts. In fact, the best possible scenario suggested that there would be potential for a 66.4% increase (from 14.9 to 24.8) in the alpha avian diversity of the baseline scenario. A more desirable management strategy would require maintaining the influencing variables at the baseline levels, and still applying restrictions on tourism activities and the prohibition of water discharges. In this case, the NBHN simulations predicted the alpha avian diversity to be 18.4 (±8.21 S.D.). But, clearly, management strategies as close as possible to the best possible scenario identified in this study are highly advisable in the coastal wetlands of Sardinia. 

## 5. Conclusions

Human impacts and climate change are reducing the ability of wetlands to provide essential services to waterbird species. The proactive management of the remaining marginal wetlands urgently requires methodological tools able to test numerous counterfactual and management scenarios, exploring their implications for the conservation of avian diversity prior to field interventions. 

In this study, we showed that simulation modeling based on non-parametric Bayesian networks can provide the necessary level of mathematical abstraction to make realistic in silico replicates of complex biological systems, thus providing a well-grounded and flexible approach to dealing with inherent uncertainties in wetland management and waterbird conservation.

Most wetlands considered in this study belong to the Natura 2000 network; therefore, the interventions proposed could be included in their management plans. 

## Figures and Tables

**Table 1:** Description of the variables used in this study.

Variable	Unit of Measure	Range	Description
Wetland size	hectares	13.3–2048	
Isolation	meters	296–54,472	
Distance to the coastline	meters	0–2050	
Mean water level	dimensionless	1–11	1 = from 0 to 10 cm; 2 = from 10 to 20 cm; 3 = from 20 to 30 cm, etc.
Water salinity	dimensionless	0–3	0 = absent; 1 = localized; 2 = scattered; 3 = widespread
Water diversions	dimensionless	0–3	0 = absent; 1 = localized; 2 = scattered; 3 = widespread
Water discharges	dimensionless	0–2	0 = absent; 1 = localized; 2 = scattered
Tourism pressure	dimensionless	0–3	0 = absent; 1 = localized; 2 = scattered; 3 = widespread
Anthropization	dimensionless	0–2	0 = absent; 1 = localized; 2 = scattered
Number of species	dimensionless	2–32	

**Table 2:** Description of the 18 scenarios simulated to predict their effects on the level of avian diversity in the 22 Sardinian wetlands under study.

Scenario Type	Code	Conditionalization	Outcome
Baseline scenario	(a)	none	the baseline level of avian diversity
Worst-case scenario	(b)	tourism pressure = 3	the expected level of avian diversity if tourism pressure becomes widespread in all wetlands
Worst-case scenario	(c)	water salinity = 3	the expected level of avian diversity if water salinity becomes widespread in all wetlands
Worst-case scenario	(d)	water discharges = 2	the expected level of avian diversity if water discharges become scattered in all wetlands
Worst-case scenario	(e)	anthropization = 2	the expected level of avian diversity if anthropization becomes scattered in all wetlands
Worst-case scenario	(f)	water level = 11	the expected level of avian diversity if water level exceeds 100 cm in all wetlands
Worst-case scenario	(g)	water diversions = 0	the expected level of avian diversity if water diversions become null in all wetlands
Worst-case scenario	(h)	scenarios *b*–*g* together	the expected level of avian diversity if scenarios from *b* to *g* occur all together
Best-case scenario	(i)	tourism pressure = 0	the expected level of avian diversity if tourism pressure becomes null in all wetlands
Best-case scenario	(j)	water salinity = 0	the expected level of avian diversity if water salinity becomes null in all wetlands
Best-case scenario	(k)	water discharges = 0	the expected level of avian diversity if water discharges become null in all wetlands
Best-case scenario	(l)	anthropization = 0	the expected level of avian diversity if anthropization becomes null in all wetlands
Best-case scenario	(m)	water level = 3	the expected level of avian diversity if water level is between 20 and 30 cm in all wetlands
Best-case scenario	(n)	water diversions = 3	the expected level of avian diversity if water diversions become widespread in all wetlands
Best-case scenario	(o)	scenarios *i*–*n* together	the expected level of avian diversity if scenarios from *i* to *n* occur all together
Mixed scenario	(p)	same as scenario *h* but tourism pressure = 0	the expected level of avian diversity if all conditions deteriorate except for tourism pressure that becomes null
Mixed scenario	(q)	same as scenario *h* but water salinity = 0	the expected level of avian diversity if all conditions deteriorate except for water salinity that becomes null
Mixed scenario	(r)	same as scenario *h* but water discharges = 0	the expected level of avian diversity if all conditions deteriorate except for water discharges that become null

## Data Availability

Data available via the Researchgate Digital Repository http://doi.org/10.13140/RG.2.2.17555.76325.
